# Developing Fiber Specific Promoter-Reporter Transgenic Lines to Study the Effect of Abiotic Stresses on Fiber Development in Cotton

**DOI:** 10.1371/journal.pone.0129870

**Published:** 2015-06-01

**Authors:** Junping Chen, John J. Burke

**Affiliations:** Plant Stress and Germplasm Development Unit, Cropping Systems Research Laboratory, Agricultural Research Service, United State Department of Agriculture, Lubbock, Texas, United States of America; USDA-ARS-SRRC, UNITED STATES

## Abstract

Cotton is one of the most important cash crops in US agricultural industry. Environmental stresses, such as drought, high temperature and combination of both, not only reduce the overall growth of cotton plants, but also greatly decrease cotton lint yield and fiber quality. The impact of environmental stresses on fiber development is poorly understood due to technical difficulties associated with the study of developing fiber tissues and lack of genetic materials to study fiber development. To address this important question and provide the need for scientific community, we have generated transgenic cotton lines harboring cotton fiber specific promoter (CFSP)-reporter constructs from six cotton fiber specific genes (*Expansin*, *E6*, *Rac13*, *CelA1*, *LTP*, *and Fb late*), representing genes that are expressed at different stages of fiber development. Individual CFSP::GUS or CFSP::GFP construct was introduced into Coker 312 via *Agrobacterium* mediated transformation. Transgenic cotton lines were evaluated phenotypically and screened for the presence of selectable marker, reporter gene expression, and insertion numbers. Quantitative analysis showed that the patterns of GUS reporter gene activity during fiber development in transgenic cotton lines were similar to those of the native genes. Greenhouse drought and heat stress study showed a correlation between the decrease in promoter activities and decrease in fiber length, increase in micronaire and changes in other fiber quality traits in transgenic lines grown under stressed condition. These newly developed materials provide new molecular tools for studying the effects of abiotic stresses on fiber development and may be used in study of cotton fiber development genes and eventually in the genetic manipulation of fiber quality.

## Introduction

Cotton (*Gossypium hirsutum* L.) fiber (lint) yield and fiber quality are two primary factors determining the economic outcomes for cotton producers and the later is also critical for textile industry [1]. About 50% of US cotton is produced in Texas and more than 25% is produced in arid region of Texas High Plains where increasingly shortage in water supply for irrigation has made cotton growers to either reduce water usage for irrigation and/or grow cotton on dryland conditions. Drought combined with sporadic heat waves in the mid growing season and low night temperatures in the early and late season not only reduces the overall growth of cotton plants, but also greatly decreases cotton yield and fiber quality such as high micronaire, short fiber length and reduced fiber uniformity etc., hence reduces the profit margin for cotton producers and the competitiveness of US cotton industry on world market.

Cotton fibers are trichomes that are differentiated from the ovule epidermis of cotton. Fiber development consists of four distinct but overlapping stages, fiber initiation, fiber cell elongation, secondary cell wall deposition, and maturation [2]. It starts at fiber initiation where certain numbers of ovular epidermal cells differentiate into potential fiber cells (fiber initiation; ± 2 days post anthesis, DPA) followed by extensive cell elongation of fiber cell (primary cell wall deposition; 0–21 DPA). During this time, the length of thin-walled tubular fiber cell extends rapidly, reaching to its maximum length. This is also the stage where cotton boll enlarges rapidly and attains its maximum volume at the end of 21 DPA. The secondary cell wall deposition starts at about 15 DPA and lasts about 3 to 4 weeks. During this time, cellulose is deposited inside the elongated fiber tube, forming the secondary cell wall of the fiber cell. At the end of this stage, the boll reaches its maximum weight and the maturation processes for fiber, seeds and boll itself begin (35–50 DPA).

The first three stages of fiber development are extremely sensitive to environmental stresses [3]. Drought, temperature extremes or nutrient deficiencies occur during fiber initiation and fiber elongation stages can significantly reduce the total number of fiber cells within a boll, the final length of the fiber, in addition to causing shedding of young squares, flowers, and young bolls, the factors directly related to yield and quality [3–5]. Stresses occur at secondary cell wall deposition stage can significantly affect fiber thickness and fiber strength, factors directly contributing to lint weight and fiber quality [6].

Studies have showed that many genetic and biological processes are involved in fiber development at various stages [7–11]. Understanding the relative importance of these processes to fiber development and the impacts of environmental stresses on them are fundamental for improving fiber quality via genetic and molecular approaches and sustaining cotton production under unfavorable conditions. Nevertheless, despite years of research efforts, progress in this area is diminutive and the response of these processes to environmental stresses is poorly understood. One of the limiting factors is the lack of molecular and genetic tools that allow researchers to quantitatively dissect the important processes in this complex system and to examine the effects of various stress factors on these processes.

**Fig 1:**
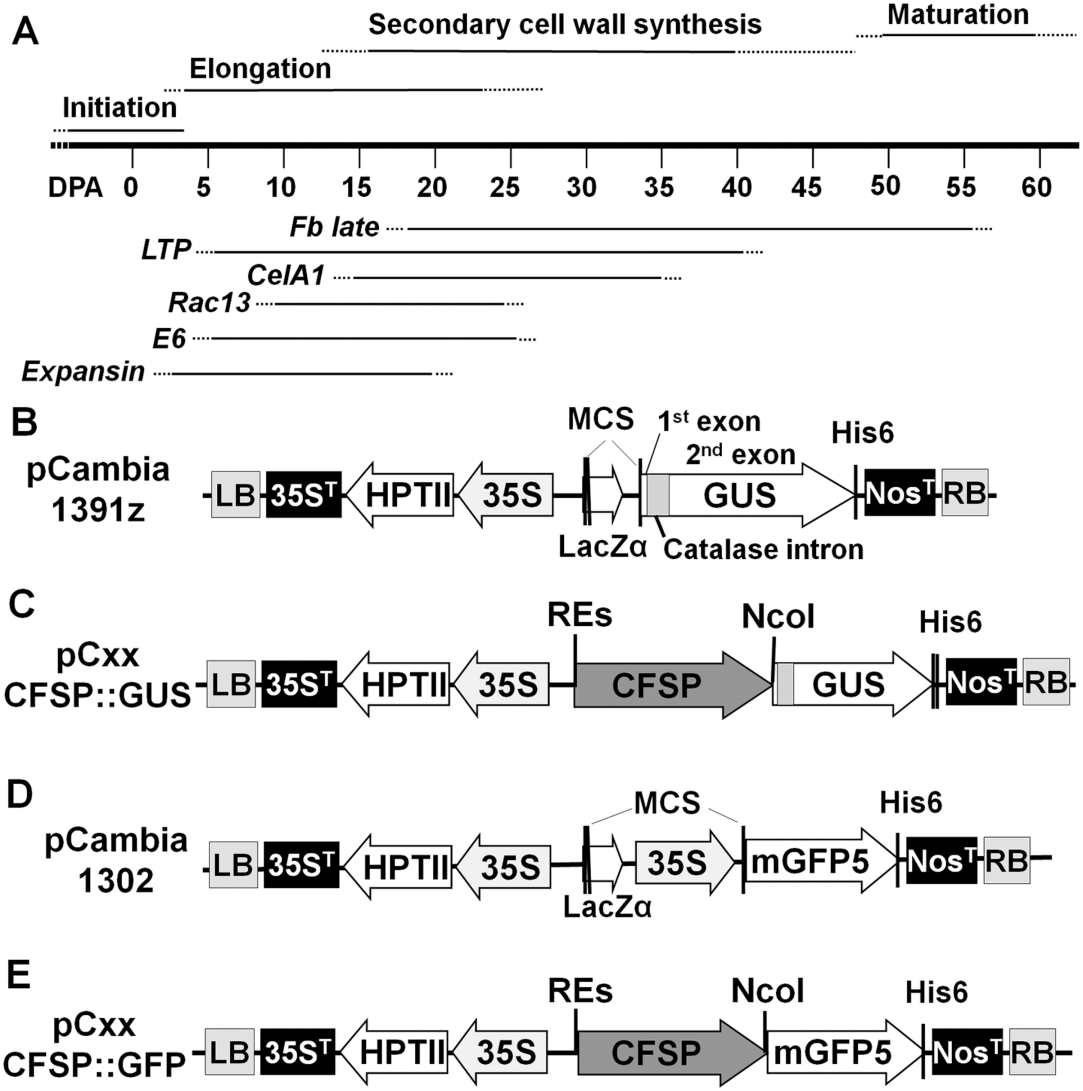
Schematic diagrams of (A) fiber developmental stages and activity of six cotton fiber specific genes during fiber development and (B-E) the pCambia binary vectors and cotton fiber specific promoter-reporter CFSP::GUS and CFSP::GFP constructs. (A) Schematic diagram showing the four developmental stages of cotton fiber and expression time frame of six corresponding cotton fiber specific genes during fiber development. All the cotton fiber specific promoter constructs contain a plant selectable marker gene cassette [cauliflower mosaic virus (CaMV) 35S::hygromycin phosphotransferase (HptII)-35S terminator (35S^t^)] adjacent to the left border (LB) and a CFSP::reporter gene-6His tagged:nopaline synthase terminator [Nos^T^] cassette adjacent to the right border (RB) of the binary vector, pC1391z and pC1302. (B) pCambia1391z T-DNA region showing the first exon, catalase intron, second exon and the His-6 tag of reporter *GusA* gene. (C) schematic diagram of CFSP::GUS constructs showing the CFSP linked to *GusA* reporter gene. (D) pCambia 1302 T-DNA region showing the CaMV 35S promoter::mGFP5-His6 structure. (E) schematic diagram of CFSP::mGFP5 showing the replacement of CaMV 35S with CFSP adjacent to *mGFP5-His6* reporter gene.

Promoter-reporter systems have been used to dissect the mechanisms of gene expression and gene regulation in plant systems. Promoter is a segment of DNA usually located upstream of a gene. It contains regulatory elements that regulate the transcription of that particular gene (when, where, how much and how long) under different internal and external conditions. Assays of reporter gene activities are used to monitor the promoter activities and quantitatively determine the kinetic changes of promoter gene product at various developmental stages and/or under diverse conditions [12–14]. In cotton, fiber specific promoter-reporter systems can be used to study the regulation of fiber genes at different fiber developmental stages and responses of fiber genes to environmental stress. In this study, we developed several different transgenic cotton lines harboring a series of promoter-reporter systems using the promoter sequence of six cotton fiber specific genes (*Expansin*, *E6*, *Rac13*, *CelA1*, *LTP*, *and Fb late)* that express at different fiber developmental stages (Fig 1A). Here we report the characterization of the cotton fiber specific promoter-reporter (CFSP-reporter) transgenic cotton lines and discuss the application of these lines as molecular tool to decipher the impacts of abiotic stress on fiber gene expression at different developmental stages of cotton fiber cells.

## Materials and Methods

### Plant materials, bacterial strains and chemicals

Cotton cultivar Coker 312 (*G*. *hirsutum* var. Coker 312) was selected as recipient to generate CFSP-reporter transgenic cotton plants. The pBluscirpt SK(+) (pBSK^+^, GI: L08787) was used to clone the promoter fragments and the pC1391z (GI:7638131) and pC1302 (GI:7638073) binary vectors (Cambia, Black Mountain, ACT, Australia) were used to make CFSP-report constructs. *Escherichia coli* strain DH5α cells were used for cloning and propagation of all the recombinant plasmid vectors. *Agrobacterium tumefaciens* strain LBA4404 was used for cotton hypocotyl transformation. Unless otherwise indicated, all chemicals and reagents used in this study were purchased from Sigma (St. Louis, MO).

### Cotton fiber specific gene promoters

Plasmid clones containing DNA fragments of promoter region of 6 cotton fiber specific genes (Fig 1A; *Expansin*, *E6*, *Rac13*, *CelA1*, *LTP* and *Fb late*) were generated by Monsanto Company (St Louis, MO) and kindly provided to Cotton Incorporated (CI; Cary, NC). The promoter fragments of different genes were embedded in the original plasmid clones in various forms along with other DNA fragment(s). After obtaining these plasmids from CI, the nucleotide sequence of each clone was sequenced from both ends, first using M13 reverse and forward universal primers followed by stepwise sequencing using designed specific primers on ABI Prism 3100 Genetic analyzer (Applied Biosystems, Inc. Foster City, CA). The sequences of each clone were assembled, restriction map constructed, length of promoter established and potential cloning sites identified using Vector NTI software (InforMax, Frederick, MD).

The CFSP DNA fragments with restriction sites compatible to binary vectors were cloned directly to pBSK^+^ by restriction digestion. For the ones that lack restriction sites compatible for binary vector, suitable cloning sites were introduced to the 5’ ends of promoter using specific primer set designed from 5’ and 3’ ends of the promoter region. The corresponding promoter fragment was PCR amplified from the original clone and cloned to pBSK^+^. The construct plasmid was transformed into *E*. *coli* DH5α cells by electroporation. Plasmid DNA of different clones was prepared using standard method and the accuracy of each resulting plasmid clone was verified by restriction digestion and sequencing. The pBSK^+^ plasmid clones with correct promoter DNA sequences were selected as donors for making CFSP-reporter constructs.

### Constructing the cotton fiber specific promoter-reporter gene cassettes

Promoterless binary vector pC1391z was used to make all CFSP::GUS constructs and pC1302 was used to make all CFSP::GFP constructs. The T-DNA region of pC1391z contains a *GusA* gene interrupted by a modified castor bean catalase intron (Fig 1B) and pC1302 contains an *mGFP5* gene (Fig 1D), both with a nopaline synthase terminator adjacent to the right border and a plant selectable marker gene, hygromycin phosphotransferase (*HptII*) driven by cauliflower mosaic virus 35S promoter at 5’ and with a CaMV 3’ UTR at 3’, adjacent to the left border.

The CFSP::GUS constructs were made by directly cloning the CFSP fragments from pBSK^+^ plasmids into the promoterless binary vector pC1391z (Fig 1C). For CFSP::GFP constructs, the original CaMV35S promoter driving the mGFP5 gene in pC1302 was replaced by a specific CFSP DNA fragment (Fig 1E). The constructs containing the proper CFSP-reporter was transformed into *A*. *tumefaciens* by electroporation.

### Cotton transformation

The CFSP::reporter cassettes were introduced into cotton hypocotyl tissues of Coker 312 via *Agrobacterium*-mediated transformation. Transgenic cotton plants were generated according to the procedures described [15,16]. Briefly, hypocotyl segments of 5 mm in length were co-cultured with *A*. *tumefaciens* on a solid medium at 28°C for 2 days followed by callus induction on a solid modified MS medium. Callus tissues formed at the ends of hypocotyl were excised and cultured in a liquid cell-suspension medium on a rotary shaker to promote embryogenesis. The resulting pre-embryo cell clusters were plated to a solid medium and the embryogenic tissues formed were transferred to solid Stewart medium for plantlet regeneration. Here, each cell suspension culture was regarded as an individual cell line and cotton plants regenerated from the same cell line are considered as an independent family.

Healthy looking regenerated cotton plants (T_0_) with 4–6 leaves and a well-established root system were transplanted to Sunshine Mix #1 potting soil (Sun Gro Horticulture Distributors Inc., Bellevue, WA) and maintained under humid and partial light conditions for a week prior to grow under normal greenhouse conditions (40–60% relative humidity, 27°C–31°C, 14h/10h photoperiod) to set T_1_ seeds. The presence of selection marker in T_0_ plant was examined by PCR amplification of a 462 bp DNA fragment of *HptII* gene from genomic DNA isolated [17]. Plants that escaped selection and yielded no *HptII* fragment were discarded. On average, 5 to 8 transgenic events were performed for each of the constructs and 5 to 10 transgenic T_0_ plants were obtained from each transformation event.

### Transgenic plant screens

Morphological characteristics of T_0_ to T_2_ transgenic plant lines were visually evaluated under normal greenhouse conditions. T_0_ plants that showed abnormal growth and/or development at any stage, such as stunt growth, sterility, compact, bushy or abnormal boll shapes and sizes, were discarded. Only the normal looking T_0_ plants were allowed to set T_1_ seeds. Similar morphological criteria were applied to screen T_1_ and T_2_ plant. The uniformity of T_2_ plants in growth and developments within each of the transgenic lines were also visually evaluated. In addition, plant height and boll development of T_4_ transgenic plants were recorded and compared with those of Coker 312 wildtype plants grown under the same conditions.

The hygromycin tolerance of T_1_ transgenic plants was examined by rooting ability of its seedlings on selection medium. Ten delinted T_1_ seeds from each transgenic line were sterilized and placed on MS-Hyg20-agar medium to germinate, 5 seeds per 0.5-L jar and 2 jars per line. Germinated seeds of transgenic line with normal expression of marker gene *HptII* were able to develop normal root system that was well-rooted into the selection medium. Three to four well-rooted T_1_ plants from each transgenic line were transferred onto soil to produce T_2_ seeds.

### Genetic Characterization

The genetic analyses for transgenic plants were performed when a batch of transformants were generated. The presence or absence of the *HptII* gene DNA fragment in T_1_ plants was examined by PCR amplification of *HptII* gene fragment. The copy number of transgene in selected T_1_ transgenic lines was examined by Southern blot. Genomic DNA extracted from young leaves of transgenic plants were digested with restriction enzymes EcoRI and HindIII, respectively, separated on 1.0% agarose gels (10 μg/lane) using gel electrophoresis (BioRad, Hercules, CA) and blotted onto nylon membranes (Hybond N+, Amersham). A 462 bp DNA fragment of *HptII* gene was labeled with ^32^P using random primers (Prime-a-Gene Labeling System Kit, Promega) and used as probe for overnight hybridization at 65°C. The blot was subsequently washed in each of the 2.0 SSC, 1.0 SSC, 0.5 SSC and 0.2 SSC solutions (all contain 0.1% w/v SDS) and visualized with a phosphorimager (Molecular Imager, BioRad).

The number of CFSP-Reporter transgene insertion in transgenic lines was further assessed in T_2_ generation by the segregation ratio of *HptII* gene PCR product in 20 individual T_2_ seedlings. Plants of single insertion transgenic lines were advanced to T_3_ generation.

The homozygosity of T_2_ plant lines within each single insertion line was examined by PCR amplification of *HptII* gene in 20 T_3_ seedlings as well as by the ability of 20 T_3_ seedlings rooting into selection medium. Homozygous T_2_ plant lines were identified by the presence of *HptII* fragment in PCR products of all T_3_ seedlings and the ability of all T_3_ seedlings to embed their root systems into hygromycin selection medium while the opposite is true for homozygous null transgenic T_2_ plant lines.

### Assessing promoter-reporter activity in fiber tissues of transgenic plant

To examine reporter gene product in cotton fiber tissues of transgenic plants, individual flowers on these plants were tagged on the day of flowering (0 dpa) and cotton bolls were hand harvested at appropriate developmental stages (Fig 1A). The reporter gene activities were visually examined by histochemical GUS staining of fiber tissues in 5-bromo-4-chloro-3- indolyl-β-d-glucuronic acid (X-Gluc) stain solution (0.1% w/v X-Gluc in 100 mM Na_2_HPO_4_/KH_2_PO_4_ pH 7.0, 10% Triton X-100) for CFSP::GUS transgenic plants or by the emission of green florescence of fiber tissues CFSP::GFP transgenic lines.

The levels of reporter gene expression in fiber tissues of T_1_ plants among different lines generated from the same construct were visually compared at a set DPA. Transgenic lines that showed normal level of promoter activities were advanced to next generation. The uniformity of reporter gene expression among T_2_ and T_3_ plants were examined in the same manner.

The promoter-reporter activity in fiber development was assessed by examining the levels of reporter gene product in cotton bolls of different DPA. For transgenic lines containing CFSP::GUS cassette, thin cross sections of cotton bolls were placed on a clean petri dish and incubated in GUS staining solution at 37°C. The GUS activity was determined by the distinct stained blue color as a result of enzymatic cleavage of X-Gluc in the presence of GUS protein in fiber tissues and the intensity of blue stain was photographed after 15, 30 and 60 min incubation. To compare the GFP levels in fiber tissues of CFSP::GFP transgenic plants, the intact cotton tissue from one chamber of harvested cotton bolls was removed and placed on the sample stand of a LT-9900 illumatool Bright Light System Epi (Illumatool Research, Encinitas, CA). The fluorescence of GFP in cotton tissue was visualized through a 515nm viewing filter after providing the excitation lighting at a 470nm.

### Quantitative determination of GUS activity

The expression levels of GUS reporter gene at different fiber developmental stages in CFSP-GUS transgenic lines were examined quantitatively by fluorometric determination. Cotton flowers were tagged on the day of flowering. At the set DPAs, cotton bolls were collected, outer skin removed, fiber tissues harvested, frozen in liquid nitrogen immediately and stored in -80°C freezer. The frozen fiber tissues were ground in liquid nitrogen using a freezer mill (model 6770, SPEX SamplePrep, Metuchen, NJ). Total proteins were extracted using the GUS extraction buffer (100 mM KH_2_PO_4_/K_2_HPO_4_ pH 7.8, 1.0 mM Na_2_EDTA, 1mM DTT, 1.0% v/v Triton X-100, 10% Glycerol) and centrifuged for 10 min (13000 rpm) at 4°C. Protein concentration in supernatant was estimated by the Bradford method [18]. Fluorometric quantification of GUS activity was performed using 4-methylumbelliferone (4-MU) substrate [19] and fluorescence was determined on Tecan 200 fluorometer (Tecan, Durham, NC) using 360nm and 465nm as excitation and emission wavelengths, respectively. The GUS activity is expressed as pmol of 4-MU per min per μg of total protein.

### Greenhouse abiotic study

Drought and heat stress study were performed under controlled greenhouse conditions using T_4_ seeds of selected C28 E6-GUS and C27 Rac13-GUS transgenic lines, respectively. Seeds from wild type Coker 312 and transgenic lines were planted into 30 cm diameter pots containing Sunshine Mix #1, 3 seeds per pot. Seedlings were thinned to one plant per pot 7 days after seedling emergence. All plants were grown under normal conditions (31°C/27°C, fully irrigated) from planting to first flower stage. After all plants entering the flower stage, the plants were randomly assigned into 2 treatment conditions, control (31°C/27°, well watered) and moderate drought stressed (C28 line), or control and moderate heat stressed (C27 line), 6 pots per line per treatment. Moderate drought stress was applied by reducing water supply by about 75% of fully watered plants. Moderate heat stress was applied by transferring the selected cotton plants to a large walk-in growth room set at temperature profile as 27°C-5 hrs, 27°C to 34°C ramping up at 1°C per hr, 34°C-5 hrs, 34°C to 27°C ramping down at 1°C per hr. Flowers were tagged each day and cotton bolls were harvested at different DPAs. GUS activity in fiber tissues was quantitatively determined using the fluorometric assay. The remaining cotton bolls were allowed to grow to maturity. Cottons were harvested at 56 DPA, ginned and delinted. Lint was analyzed by high volume instrument method at the Fiber and Biopolymer Research Institute, Texas Tech University (http://www.fbri.ttu.edu/fbri_index.php) and compared with those of the control plants.

### Bulk seed production in the field

To increase the seed supply, T_3_ seeds produced from selected homozygous transgenic and null plant lines were planted in the USDA-ARS Cropping Systems Research Laboratory field at Lubbock location (N33°35.5, W 101°53.9) in 2008 (C19-C28) and in 2009 (all transgenic lines), 6 inch apart in 4 five-meter rows for each transgenic line, along with Coker 312. Each year, permission for growing transgenic cotton lines at the USDA-ARS research field was obtained from APHIS before planting. Independent transgenic lines generated from the same construct were planted in adjacent rows. Lines generated from different constructs were separated by a 5-feet-wide row space. Sorghum was planted as a 40-ft wide field borders and in the surrounding fields. During the growing season, the reporter gene activities in 12 individual T_3_ plants from each line were examined to ensure the homozygosity of transgenic and null lines. Cottons from the 2 inside-rows plants were hand-harvested at the end of growing season and ginned. Seeds were acid-delinted, screened for quality, and stored in a seed-storage room. Remaining cottons in the field were machine harvested, ginned and seeds were autoclaved and then discarded according to APHIS’ guidelines.

## Results and Discussion

### Eleven CFSP-reporter cassettes were constructed for 6 cotton fiber specific promoters

The 6 promoters used in this study were initially isolated from cotton fiber specific genes that express at different fiber developmental stages (Fig 1, S1 Table). The *Expansin* and *E6* genes express mostly at fiber cell elongation stage [20,21]. The *Rac13* expresses at late cell elongation to mid secondary cell wall deposition stages [22]. *Fb* late (4–4) and *CelA1* (cellulose synthase A1) express mostly at secondary cell wall deposition stage [23,24] while *LTP* (lipid transfer protein) expresses at the first three fiber developmental stages (PCT/US96/09897). The nucleotide sequence of each promoter is provided as supplemental materials (S1 Text).

The detailed information about the 11 constructs is presented in Table 1. A total of 8 CFSP::GUS constructs were made for the 6 CFSPs. One CFSP::GUS construct was generated for each of the *Expansin*, *Rac13*, *LTP*, and *CelA1* promoters. Two CFSP::GUS cassettes were constructed for each of the *E6 and Fb-late (4–4)* promoters using DNA fragments of different length, one with full length promoter fragment in original clone and the other one with fragment shortened from the 5’-end. Three CFSP::GFP constructs were generated using *CelA1*, *Fb late (4–4)* and *LTP* promoter fragments, respectively.

**Table 1:** Information about CFSP::Reporter constructs and transgenic lines.

Transgenic line	CFSP::reporter constructs	Promoter length (bp)	Cloning sites	No of T_0_	Lines for Distribution
C19	E6-short::GUS	1186	EcoRI-NcoI	18	2
C28	E6::GUS	2640	PstI-NcoI	54	4
C21	CelA1::GUS	2898	PstI-NcoI	86	4
C22	Expansin::GUS	2192	NotI-NcoI	28	5
C25	LTP::GUS	2983	PstI-NcoI	62	5
C27	Rac13::GUS	4013	PstI-NcoI	54	5
C33	Fb late::GUS	4124	BamHI-NcoI	8	3
C34	Fb late-short::GUS	2422	PstI-NcoI	11	2
C30	CelA1::GFP	2898	PstI-NcoI	47	4
C31	LTP::GFP	2983	PstI-NcoI	33	4
C32	Fb late::GFP	2422	PstI-NcoI	8	4

### Transgenic line characterization

Multiple transformation events were performed in developing the transgenic cotton lines evaluated in this study. A total of 409 confirmed independent T_0_ cotton transgenic lines were generated, 321 for the 8 CFSP::GUS constructs and 88 for the 3 CFSP::GFP constructs (Table 1). Plants of transgenic lines were subjected to meticulous genetic and morphological evaluation from T_0_ to T_3_ generations under controlled laboratory and greenhouse conditions. In general, the T_3_ plants of selected transgenic lines were morphologically uniform and similar to Corker 312 wildtype plants in the growth and development.

Visual examination of GUS staining and GFP fluorescence of T_1_ transgenic lines of the same construct showed some variations in the intensity among different lines of the same construct, indicating amendment of promoter activities by genetic background of host plant lines. Since one of the goals of this study was to develop a set of CFSP-reporter transgenic cotton lines for molecular studies of gene regulation during fiber development, we selected only those transgenic lines that showed normal level of promoter activities (comparing among all lines) in T_1_ plants and advanced them to in T_2_ generation. In general, any transgenic line that was *HptII* ^+^ (checked by PCR) and morphologically normal, but showed weak expression of reporter gene (comparing with other lines) was discarded (Table 2).

**Table 2:** Segregation of T_2_ seedlings of selected C30 and C31 cotton transgenic lines for rooting on hygromycin-selection medium and for the presence/absence of hygromycin DNA fragment in PCR products.

Transgenic line, T_1_	T_1_ fiber GFP fluorescence	T_2_ rooting Yes-no	T_2_ PCR screen *HptII* ^+^/*HptII* ^-^	Estimated insertion no	Advance to T_3_
C30-10-3, P3	High	12–8	15–5	1	Yes
C30-11-7, P1	Moderate	20–0	18–2	>1†	No
C30-12-1, P2	Moderate	13–7	15–5	1	No
C30-19-4, P1	High	16–4	15–5	1	Yes
C30-23-1, P3	High	13–7	14–6	1	Yes
C31-3A-1, P2	High	14–6	13–7	1	Yes
C31-9-3, P1	High	14–6	14–6	1	Yes
C31-11A-3, P1	Good	19–1	19–1	>1	No
C31-12A-6, P3	High	15–5	16–4	1	Yes
C31-14-3, P3	High	16–4	15–5	1	Yes
C31-19-7, P1	Weak‡	NA§	NA	NA	NA

^†^: Transgenic line contains 2wo or more copies of CFSP-reporter transgene;

^‡^: This transgenic line was discarded due to weak expression of the reporter gene;

^§^: Not applied. No T2 seeds were harvested from this line.

The genetic characterization of transgenic plants was performed in batches as the transgenic lines were developed. As illustrated in S1 Fig, Southern blotting analysis of T_1_ genomic DNA indicated that the majority of the transgenic lines contained a single copy of CFSP-reporter transgene. Genetic analysis of T_2_ plants for segregations of *HptII* ^+^/ *HptII* ^-^ and rooting on hygromycin selection medium yielded results similar with those of Southern blot. Table 2 exhibited the segregations of T_2_ seedlings in one of the genetic screens. Nine out of eleven selected C30 (CelA1::GFP) and C31 (LTP::GFP) transgenic lines had a segregation ratio of about 3 to 1 for *HptII* ^+^/*HptII* ^-^ and rooting/none-rooting on selection medium among T_2_ plants, indicating single insertion of transgene in these lines. Two other lines, C30-11-7-1 and C31-11A-3, had a segregation ratio much higher than 3 to 1 for hygromycin tolerance, suggesting the likelihood of two or more copies of transgenes in these 2 lines. As mentioned above, the C31-19-7 line was discontinued at T_1_ generation due to the weaker than normal GFP levels presence in cotton fibers.

**Fig 2:**
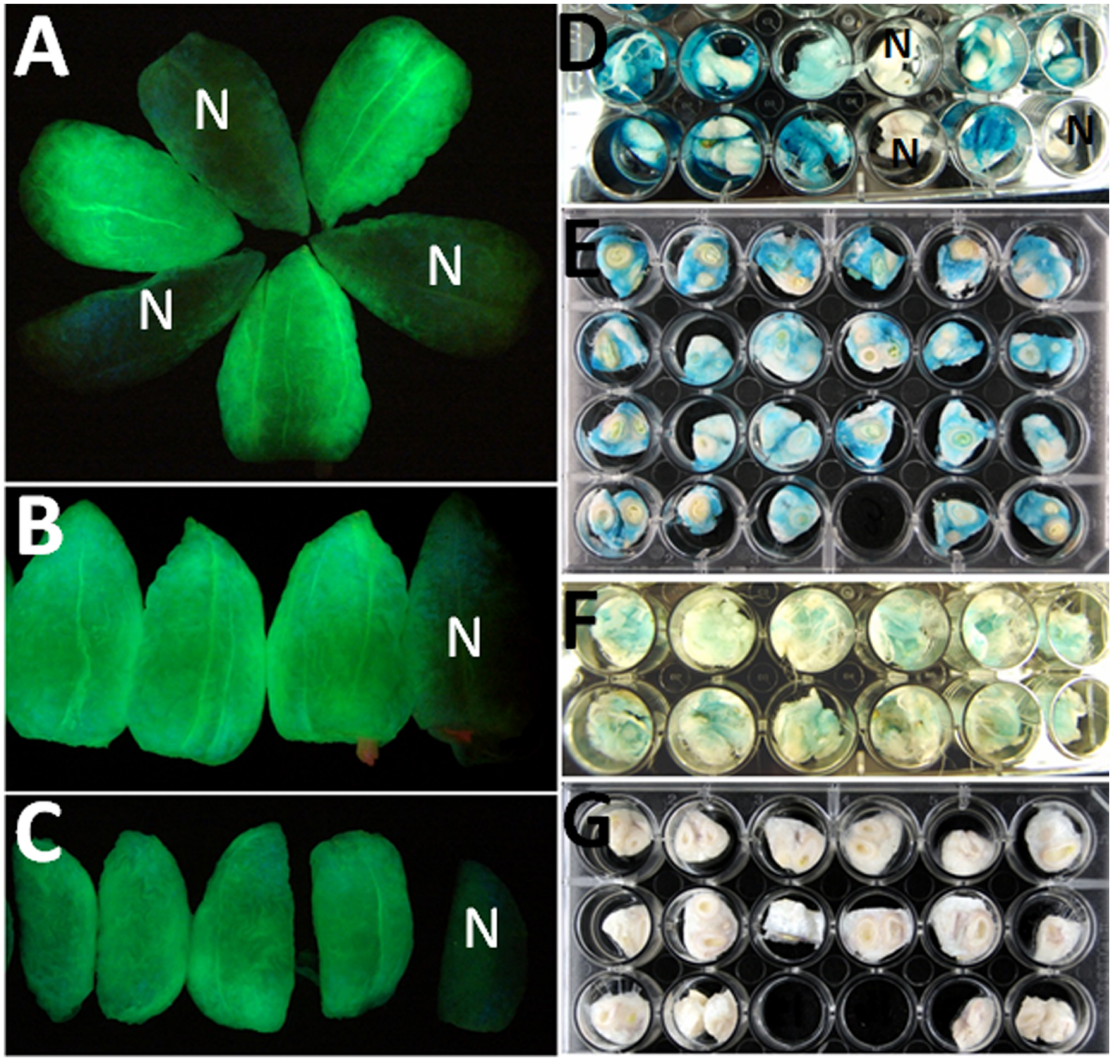
GFP fluorescence and GUS histochemical staining showing the expression of reporter genes in cotton fiber tissues of CFSP::GUS and CFSP::GFP transgenic plants. GFP fluorescence displayed by the transgenic cotton fiber tissues of (A) C30 (CelA1::GFP), (B) C31 (LTP::GFP) and (C) C32 (Fb late::GFP) transgenic plants. The dark, non- fluorescence fiber tissues were from null transgenic plants (labeled N). D-G, GUS histochemical staining of cotton fiber tissues harvested from individual transgenic plants of: (D) a T_2_ family showing the segregation of reporter gene; a T_3_ family from a homozygous transgenic T_2_ plant showing uniform and different levels of GUS gene expression at (E) mid and (F) later fiber developmental stages; (G) a T_3_ family from a homozygous null transgenic T_2_ plant showing no Gus gene activity in any of the T_3_ plants.

Consistent with molecular and genetic analysis, the GFP fluorescence (Fig 2B and 2C) and GUS staining (Fig 2D) of T_2_ plant fibers showed segregation of reporter activity similar to those of PCR amplification of *HptII* gene and rooting on hygromycin selection medium. Also, true homozygous T_2_ plant lines identified by genetic segregation showed uniform GUS production in fiber tissues of all T_3_ plants (Fig 2E and 2F) while no GUS activity was detected in null transgenic T_2_ (Fig 2D, labeled with N) and T_3_ plants (Fig 2G). In summary, after vigorously genetic and morphological screening, a total of 51 independent transgenic lines were selected and advanced to T_4_ generation, about 4 to 6 independent GUS or GFP transgenic lines for each of the 6 CFSPs (Table 1).

**Fig 3:**
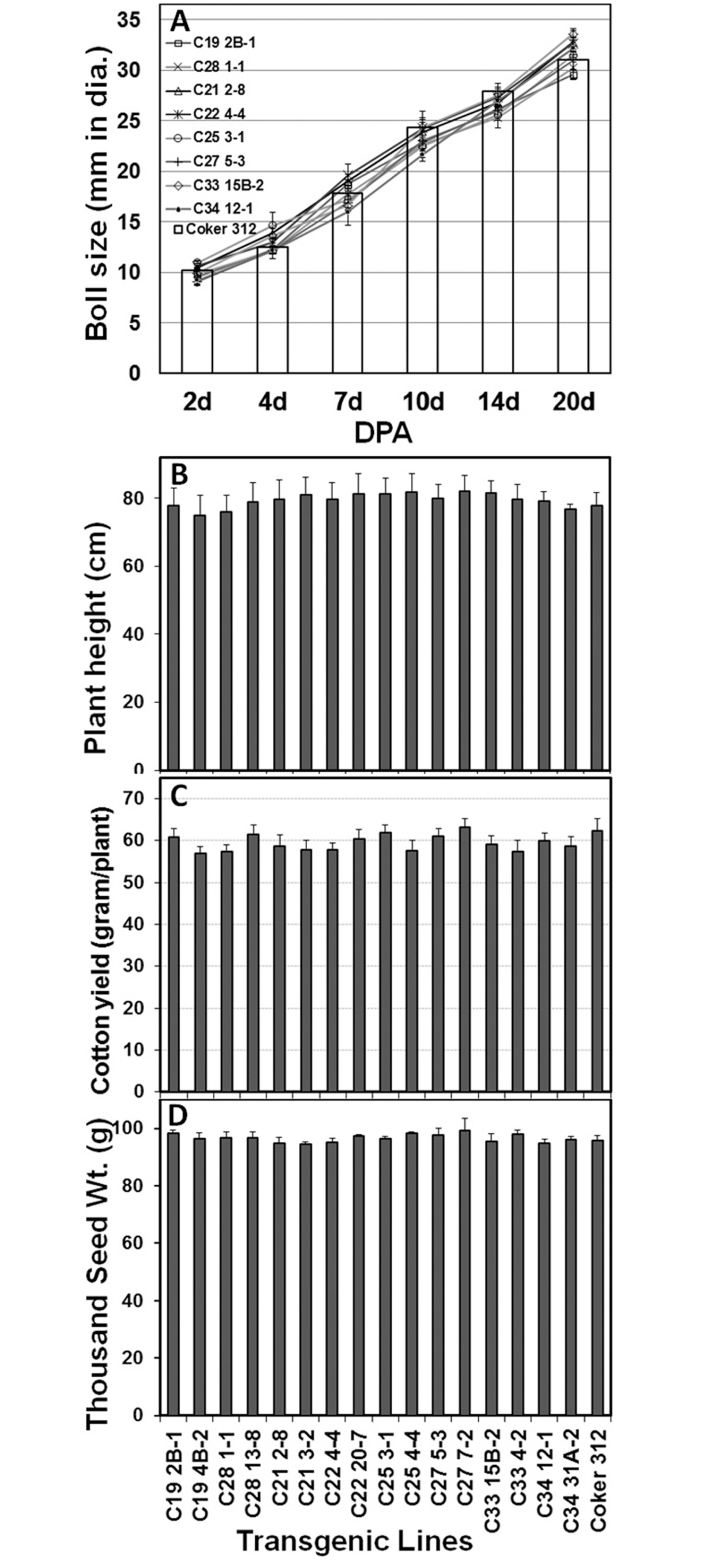
Comparisons of agronomic traits of T_4_ CFSP::GUS transgenic lines with those of Coker 312 wildtype plants under normal conditions. **(A)** cotton boll size, Coker 312: open column, transgenic lines: lines. (B) Plant height. (C) Cotton yield per plant. (D) Delinted 1000-seed weight.

In addition to molecular and genetic characterization, we also measured and compared the agronomic traits of transgenic plants with those of Coker 312 wildtype plants under normal greenhouse and field conditions. Fig 3 showed the boll size, plant height, cotton yield and seed weight of representative transgenic lines of 8 CFSP-GUS constructs measured in 2009 field studies. Similar results were obtained for other transgenic lines. No significant difference was detected between transgenic and Coker 312 plants for parameters measured. Both the transgenic and null plant lines exhibited similar seed germination rate, time to first square, time to first flower, number of boll set, boll development and size, seed number per boll, seed weight and lint weight as Coker 312 plants under both greenhouse and field conditions.

### Promoter activities in CFSP-reporter transgenic plants behave similarly to its native state

**Fig 4:**
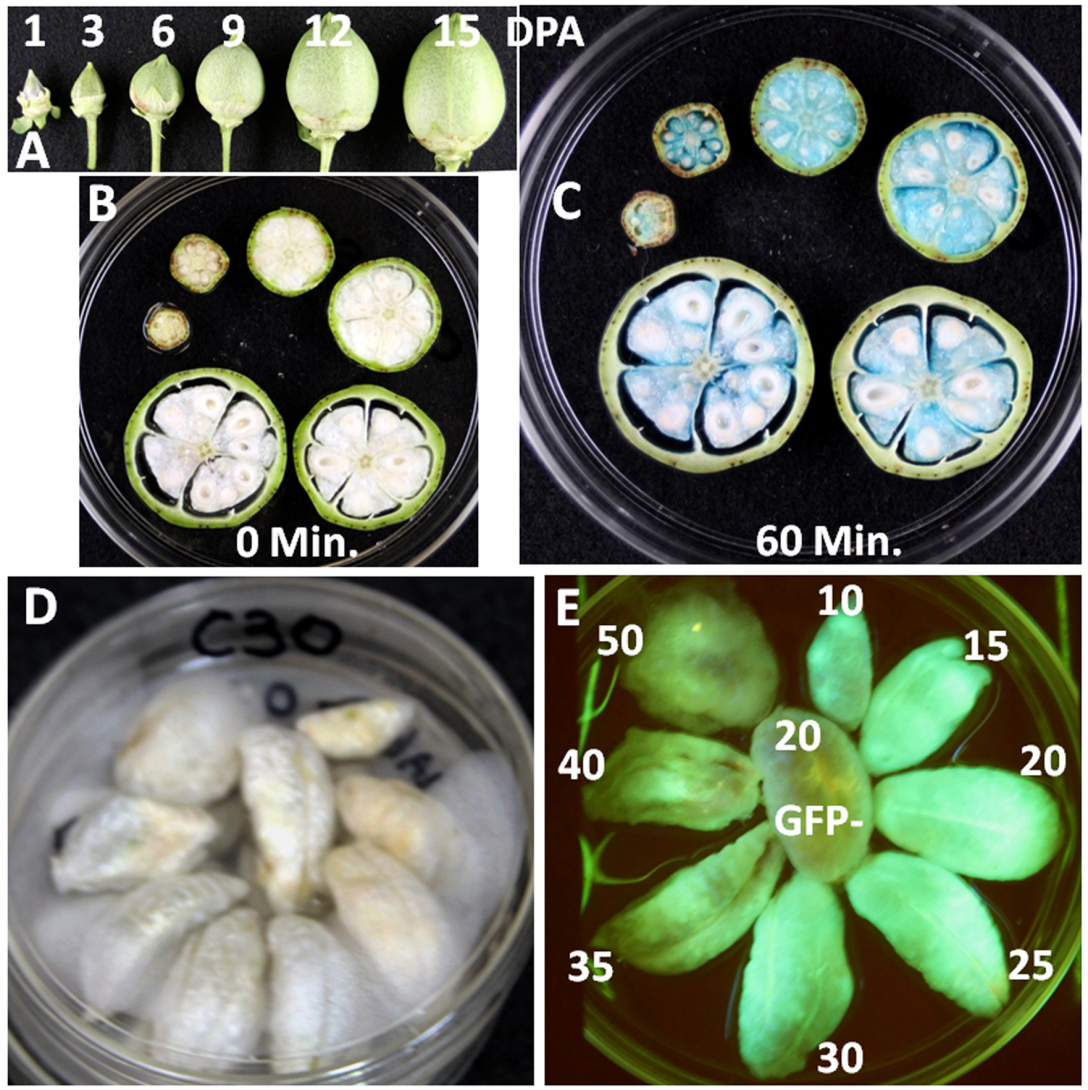
Photographs of cross-sections of cotton balls and fiber tissues harvested at different days post anthesis (DPA), showing the expression patterns of reporter gene activities in C22 (Expansin::GUS) and C30 (CelA1::GFP) transgenic plants. (A) photo of cotton balls harvested from a transgenic plant line at 1, 3, 6, 9, 12 and 15 DPA; Thin cross sections of those C22 cotton bolls in A, showing the fiber tissues (B) before GUS staining and (C) after 60-min GUS staining. (D) Fiber tissues harvested from one chamber of cotton bolls of a C30 transgenic plant line at 10, 15, 20, 25, 30, 35, 40, 50 DPA and (E) the Green-Fluorescence-Protein glowing, showing the cellulose syntheses A1 gene activity during cotton fiber development; fiber tissue at the center was from a 20-DPA boll of a null plant, serving as a GFP negative control.

**Fig 5:**
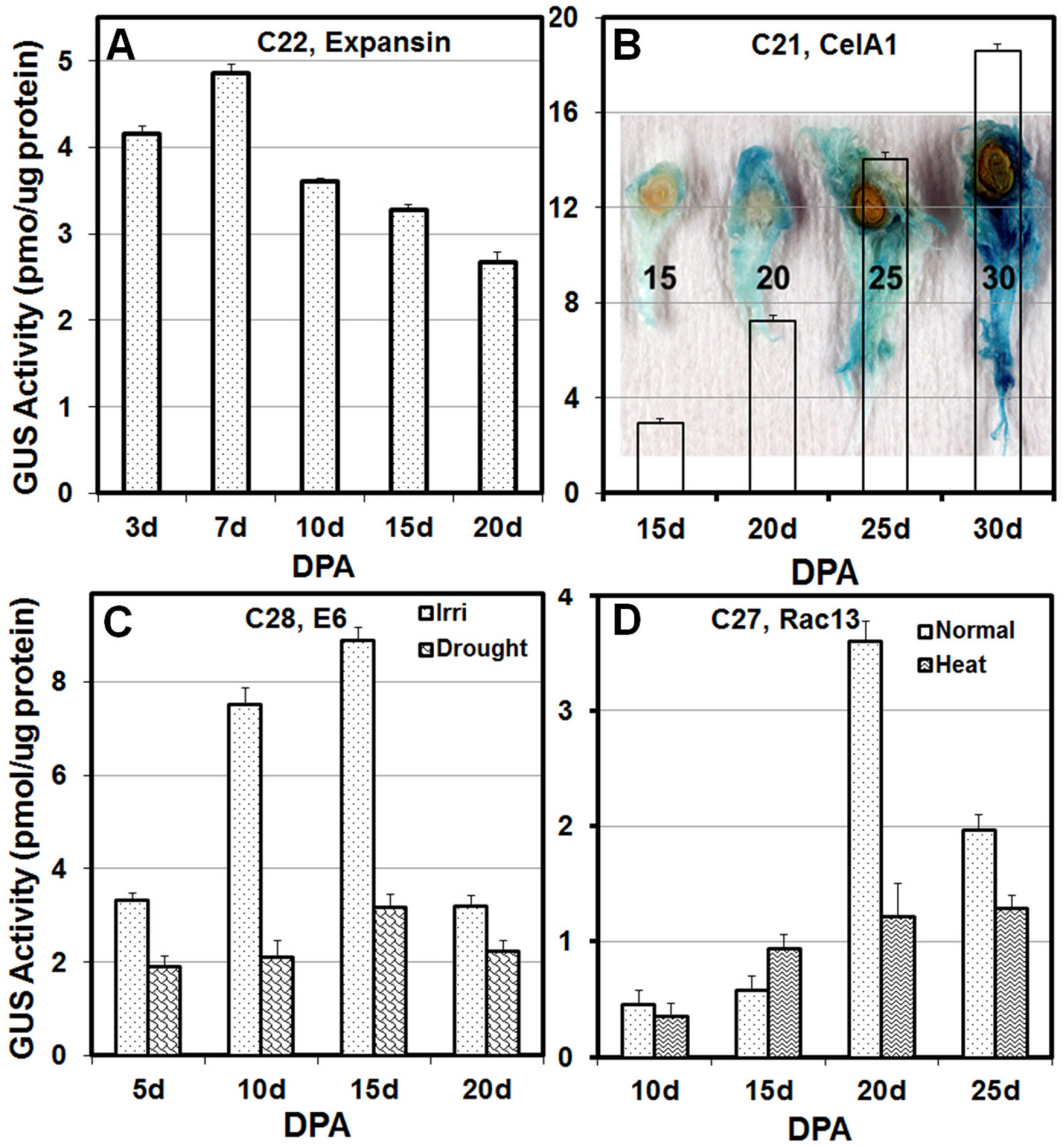
GUS activities measured by fluorometric quantification at different fiber developmental stages in transgenic plants. (A) C22- *Expansin*::*GUS*, (B) C21-*CelA1*::*GUS*, (C) C28-*E6*::*GUS* and (D) *Rac13*::*GU*S grown under normal or stressed conditions. The GUS activities were determined by measuring fluorescence on Tecan 200 fluorometer using 360nm and 465nm as excitation and emission wavelengths, respectively and expressed as pmol of 4-MU per min per μg total protein.

Promoter-reporter systems are often used to dissect the regulation of gene expression and monitor gene activities quantitatively in plant system [13]. In this study, the expression patterns of reporter gene in fiber tissues of transgenic lines were first examined by GUS staining and/or GFP fluorescence of fiber tissues at different fiber development stages followed by quantitative measurement of GUS activities at different DPAs. Fig 4 showed the visual observation of the reporter activities in expansin-GUS (C22) and CelA1-GFP (C30) systems, illustrating the changes in expansin and CelA1 promoter activities during fiber development. Studies have showed that the expansin gene was expressed mostly at fiber cell elongation stage [21] while the *CelA1* gene was expressed mostly at secondary cell wall deposition stage [23]. Our results indicated that the expansin gene was most active at the very early stages of fiber development (Fig 4C) while the CelA1 gene expressed the most at the peak of secondary cell wall deposition (Fig 4E). Quantitative measurement of GUS activity showed the kinetic changes of CFSP activities in transgenic plants (Fig 5) that were consistent with our visual observations (Fig 4) and as well as those previously reported [20–23]. The *GUS* reporter gene under the control of the expansin or E6 promoter expressed the most at early fiber development stage (Fig 5A and 5C). Expansin promoter was most active during the early phase of fiber cell elongation (Fig 5A) while E6 promoter was most active in the mid cell elongation phase (Fig 5C). The maximum activities of *Rac13* promoter occurred at about early stage of secondary cell wall deposition (Fig 5D) while the transgenic cotton plants of CelA1::GUS showed the highest reporter activity at the peak of secondary cell wall deposition (Fig 5B).

In addition to the 6 original promoters isolated, we also analyzed a short version of promoters for *E6* and *Fb late* genes (Table 1). GUS staining and quantitative measurements analyses showed no difference in expression level or tissue specificity between the short and long promoters. Studies have shown that most of the key regulatory elements are resided within the 1000 bp upstream of the starting codon of the corresponding gene [25,26]. The lengths for E6 and Fb late short promoter were 1186 and 2422 bp respectively, compared to the 2649 and 4124 bp of the original promoters.

In general, the expression patterns of *GUS* gene in different CFSP::GUS cotton transgenic lines (Fig 5) were consistent with those of original CFS genes (Fig 1) from which the promoter fragment were isolated. The results indicate that promoter fragments in CFSP-reporter transgenic plants behaved similarly to its native state [20–23]. Hence, the activities of the reporter genes in transgenic lines generated in this study represented the corresponding fiber gene activities in fiber tissues and can be used to examine the dynamic changes of different classes of fiber gene activities at different stages of fiber development.

### Impacts of abiotic stresses on fiber gene activities in CFSP-reporter transgenic lines

It is well documented that abiotic stresses, such as drought and/or high temperatures, not only cause significant cotton yield loss but also reduce fiber quality [3,4]. However, little is known about the regulation processes (traits) that are critical to the formation of high quality fiber and the association of these traits with abiotic stress tolerance in cotton. The inherent technical difficulties associated with the study of fiber tissues and the lacks of suitable molecular markers to monitor fiber development, are two of the major factors hindering the studies in this area. The CFSP-reporter system described in this study provided a set of genetic materials for examining the influence of abiotic stresses on fiber gene activities during fiber development at molecular level. E6 is one of the major fiber specific genes expressed predominantly during the mid-late cell elongation and early secondary cell wall deposition stages [20,27]. In this study, we selected C28 E6-GUS transgenic lines as an example to study the effect of drought stress on gene expression during fiber elongation. Morphologically, the water stressed cotton plants showed moderate leaf wilting phenotype in the afternoon but were able to recover somewhat the next morning. The results showed that drought stress significantly reduced E6 promoter activities throughout the entire fiber elongation phase (Fig 5C), indicating the potential negative effects of drought stress on fiber elongation.

Rac13 encodes a small GTP-binding protein. It is proposed that *Rac* genes play a key role in regulation of cytoskeletal organization [22]. The *Rac13* is predominantly expressed during the mid to late fiber elongation and early-mid secondary cell wall deposition stages [22,28]. Here we used C27 Rac13-GUS transgenic lines to examine the effect of moderate heat stress on gene expression during critical stage for fiber elongation and secondary cell wall biosynthesis. Result showed that moderate heat stress caused significant reductions in Rac13 expression in fiber tissues of 20 to 25 DPA, a period that *Rac13* is most active and fiber development has entered secondary cell wall deposition stage (Fig 5D). Nevertheless, a slight increase in *Rac13* promoter activity in heat-treated plants was noticed at the onset of the transitional phase from cell elongation to secondary cell wall deposition (15 DPA). This could be the effect of increased heat unit on the development of fiber cell as it is known that temperature affects the timing of the phase transition and fiber cell elongation [4,29,30].

Furthermore, we analyzed the quality traits of mature fibers harvested from the transgenic lines grown under normal, moderated drought and heat stressed conditions. Statistical analysis showed no significant difference for major fiber quality traits among transgenic lines grown under normal condition (Table 3). The values of micronaire, fiber length, fiber strength and fiber elongation all fall into the normal range of industrial standard (http://www.cottoninc.com/fiber/quality/Standards/). However, noticeable increases in micronaire and decreases in fiber length were observed for cottons grown under both moderate drought and heat stressed conditions (Table 3). The reductions in fiber length under stressed condition were correlated with decreases in reporter gene activities in stressed cotton fibers detected at elongation and cell wall deposition stages. Our results in greenhouse study are consistent with the field observations where plants produce shorter fibers with high micronaire reading after experiencing drought or high temperature stress episodes [3,4]. Increase in fiber strength for cottons produced under heat stressed condition was also observed. Study has showed that cottons that enter the phase transition earlier often produce fiber with higher fiber strength than those that enter the phase at normal time (Hinchliffe et al., 2011).

**Table 3:** High Volume Instrument analysis for major fiber quality traits of mature fibers harvested from greenhouse grown cotton lines under normal and stressed conditions.

Lines	Treatment†	Micronaire	Fiber length (inch)	Length uniformity index	Fiber strength	Elongation
C28-13-8	Normal	4.80	1.26	84.60	32.10	6.80
C28-13-8	Drought	5.18	1.17	85.90	32.80	6.20
C28-13-8	HT	5.48	1.11	84.50	36.20	6.00
C27-7-2	Normal	4.45	1.31	86.60	33.10	6.80
C27-7-2	Drought	5.14	1.17	83.70	33.70	6.10
C27-7-2	HT	5.47	1.16	85.60	35.00	5.60

^†^The data is the average of 3 replicates for normal and drought stress treatments and the measurement of pooled fiber sample from 3 HT-replicates due to limited boll set on HT-stressed cotton plants.

The ultimate goal of this study is to use this set of transgenic cotton lines we developed in this report to study the molecular response of fiber cell to various biotic and abiotic stresses at different fiber developmental stages and their association with fiber properties and fiber yield. The 6 promoters used in this study represented different groups of genes that are important for fiber development at the first three critical stages. The results of this study demonstrated the suitability of CFSP-reporter transgenic lines to be used in quantitative studies on fiber development genes at different fiber developmental stages. These fiber specific promoters may eventually be used in study of the genetic manipulation of fiber quality traits.

## Supporting Information

S1 Fig Southern blot of individual C19, C21, C22 and C28 T_1_ transgenic plant lines regenerated from transformation event 3. (TIF)

S1 Table Expression patterns of fiber specific promoter genes. (DOCX)

S1 Text Nucleotide sequence of cotton fiber specific promoters. (DOC)
