# Supplementary figures and images for: Developing Fiber Specific Promoter-Reporter Transgenic Lines to Study the Effect of Abiotic Stresses on Fiber Development in Cotton
Source: PLoS One. 2015 Jun 1;10(6):e0129870. doi: 10.1371/journal.pone.0129870 (PMC4451078; doi:10.1371/journal.pone.0129870)

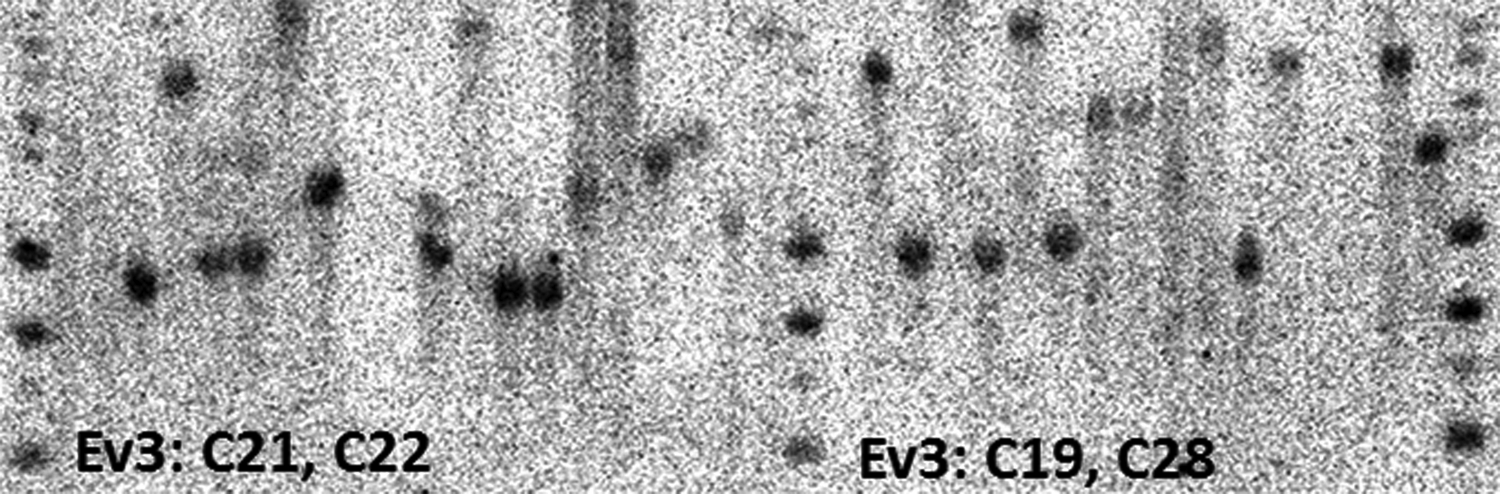

Supplement: S1 Fig — (TIF) [file pone.0129870.s001.tif]
