# Supplementary material for: Developing Fiber Specific Promoter-Reporter Transgenic Lines to Study the Effect of Abiotic Stresses on Fiber Development in Cotton
Source: PLoS One. 2015 Jun 1;10(6):e0129870. doi: 10.1371/journal.pone.0129870 (PMC4451078; doi:10.1371/journal.pone.0129870)
Supplement: S1 Table — (DOCX) [file pone.0129870.s002.docx]

**S1 Table. Expression patterns of fiber specific promoter genes.**

| **Gene of the promoter** | **Gene expression** | |
| --- | --- | --- |
|  | **At stages of fiber develop.** | **Expression level** |
| ***Expansin*** | **Early to early-mid** | **High** |
| ***E6*** | **Early to mid** | **Fairly High** |
| ***Rac13*** | **Late-Early to Mid** | **Moderate** |
| ***CesA1*** | **Mid to early-Late** | **High** |
| ***LTP*** | **Early to early-Late** | **High** |
| ***Fb late (4-4)*** | **Mid to Late** | **High** |
