# Supplementary material for: Developing Fiber Specific Promoter-Reporter Transgenic Lines to Study the Effect of Abiotic Stresses on Fiber Development in Cotton
Source: PLoS One. 2015 Jun 1;10(6):e0129870. doi: 10.1371/journal.pone.0129870 (PMC4451078; doi:10.1371/journal.pone.0129870)
Supplement: S1 Text — (DOC) [file pone.0129870.s003.doc]

**S1 Text. Nucleotide sequences of cotton fiber specific promoters.**

**E6 promoter in C19 transgenic lines**

ID E6 Promoter; EcoR1-NcoI fragment; DNA; 1186 bp.

SQ Sequence 1186 BP; 464 A; 128 C; 153 G; 441 t;

ggaattctcc tatttttatt tatttatttg cttaggaagt tttactgaca ctgcttttat 60

ttttccatca atcaaattta agagacaatt cactttttat aattaacaaa aaaaaacaaa 120

aagaaaataa aagaaattac ttttttcttt ttcgtgttcg atacaagata gatgaaatat 180

gaaaaataaa atgaaatgaa aatatattac tagtgatata tgacctccat tatgtagggg 240

aaagaaataa aaattatatt aatttatgat acttccataa tgtggttaaa aataattatc 300

tagtattttt ttgtaaaaaa aaaaaaagtt gatatctatg ctactaatga ggtttcttag 360

tgagtttgtt actactaata aagtttattt gcatggttga gaccttatgc ttttcaaata 420

cccatatttg aattttaaaa attgtgaatt tttattatat ttaaaaaaca agttatttat 480

ataactagta atgtattatt ttgacttttt tttaatcgag ttaatgttgg ttatttcgtt 540

ataccaattc aataaaatat tttatttata ttaaattata gcatacctca cgatgtgggt 600

gaagtaaaat tatttaacaa atatattttg aaaaattgat aaaaatacta aatgaggttt 660

tggttgaata gtaagatata attattacaa attataaata tgtaggttca aaatctatca 720

tgtgtatatt tgtactatta ttctatataa attgataacc ttataaaagt atctaattta 780

gtttatggtt gattgatcga taataccaaa tttattaaaa attaatatta gtaaagatat 840

atagtacaaa actaaacata aaattttata tgttaaggaa atagcggaaa aaatatcata 900

tttgtagaac tgtttagcag tgtgggagaa tgggatcatt acaaggaaaa atgaaatata 960

tatcattaat accaaacata aaagaaagcg tcttttgata aagttgttat tggtgtaatg 1020

tgaagggacc acaatcatca ccattcacca cttgctccta attgagttga aatcttttta 1080

caacatagaa aactagaaga tcgccctttc ttgcttcata tatatagatt ttgtatcatc 1140

gcaatttcac atcacacaca caagtaaagc attagcaacc atagcc 1186

**E6 promoter in C28 transgenic lines**

ID E6 Promoter; Pst1-Nco1 fragment; DNA; 2640 bp.

SQ Sequence 2640 BP; 965 A; 313 C; 375 G; 987 t;

ggtcaacgga tcaacattca attacaataa agagtataga tagatacatc aatactatcc 60

agccctcttt catgatgatg ttgcaagttt tgcactccac ccccatatta ttatgaagag 120

gagaaattcc tgttacaaca ttaattgagc tttattttct aagaaatgac ttctaacatt 180

aataaatttg aatcaattta tagctatttc tcgtactctt tcgtagtata gctttctttt 240

tatgctaaaa caggagatga ttataagacg tgaagttgtc ttaagattaa caaggcagtt 300

agatgcattt aaattggtat ttaaggatat gtctgtatat aactagaact aattaagatt 360

acaatacact gcaaaatgat tccacctttt tttttttttt ggttctctct tttatatgat 420

caaatacaat gttgatatga acaaggtttt gcagttgtag aaaatcgtgg aggacttttt 480

ttttaaaaaa agaaagataa aattcataaa aaaatgtgaa gttaagcata tttagtgatg 540

ggtggggtat ggggtggttt gctaacatgg aatgcgcatg gcagattggc actttaaaga 600

agggatgggg ccgagggggc gggagttgtt aaatcctcgg cgtagaaaaa ggtcagtaag 660

cgtgtccctg gcattagaca aggagagggg gtagcacatg cacagcccaa ctatatctct 720

ttattttatg tcccactcca ctccccctca tctctgccgc aacattaaat accttatgca 780

tccttactat tcataatggt tttttttgtt gggttgatgt tacaaaatta aattttttat 840

tatagatgta ttaatatttt taaaaatata taagattttt tttaagtagg agtttaatct 900

ttggtggtga tgttgatttt agatatattc cccacccgca agtagatatc atacattcta 960

atataattta aaaaaaagta taactaaaat atattttata tttttttata tttttttgaa 1020

tttttaaatt ttaaaaaatt aattaaatgt tcatgtgtca tctacatgta tgcaacgtta 1080

gcgaagttta aaatatatta attttttcat tcgtgatttg aaaaaaaaaa ggtaagttta 1140

aaggttaaac aagcataaat ctaaataaat agttaaaata atttttttta taaagttaga 1200

gaattaaata aattattatt ttatttaaaa ataattttca ataaattact aatttagtca 1260

cataatctaa tataatttaa aaaataatta ttaacatttt aatttgtatg aattcttgtt 1320

atgtatggat tcaaacccgt tcgttccatc aacaaattga tctgcatgag acttaagggt 1380

tagaattttg tgtaaccctt tttcctccta atttttactt ttaaaaagaa attgcaatac 1440

aatttttttt ttatagaatt ctcctatttt tatttattta tttgcttagg aagttttact 1500

gacactgctt ttatttttcc atcaatcaaa tttaagagac aattcacttt ttataattaa 1560

caaaaaaaaa caaaaagaaa ataaaagaaa ttactttttt ctttttcgtg ttcgatacaa 1620

gatagatgaa atatgaaaaa taaaatgaaa tgaaaatata ttactagtga tatatgacct 1680

ccattatgta ggggaaagaa ataaaaatta tattaattta tgatacttcc ataatgtggt 1740

taaaaataat tatctagtat ttttttgtaa aaaaaaaaaa agttgatatc tatgctacta 1800

atgaggtttc ttagtgagtt tgttactact aataaagttt atttgcatgg ttgagacctt 1860

atgcttttca aatacccata tttgaatttt aaaaattgtg aatttttatt atatttaaaa 1920

aacaagttat ttatataact agtaatgtat tattttgact tttttttaat cgagttaatg 1980

ttggttattt cgttatacca attcaataaa atattttatt tatattaaat tatagcatac 2040

ctcacgatgt gggtgaagta aaattattta acaaatatat tttgaaaaat tgataaaaat 2100

actaaatgag gttttggttg aatagtaaga tataattatt acaaattata aatatgtagg 2160

ttcaaaatct atcatgtgta tatttgtact attattctat ataaattgat aaccttataa 2220

aagtatctaa tttagtttat ggttgattga tcgataatac caaatttatt aaaaattaat 2280

attagtaaag atatatagta caaaactaaa cataaaattt tatatgttaa ggaaatagcg 2340

gaaaaaatat catatttgta gaactgttta gcagtgtggg agaatgggat cattacaagg 2400

aaaaatgaaa tatatatcat taataccaaa cataaaagaa agcgtctttt gataaagttg 2460

ttattggtgt aatgtgaagg gaccacaatc atcaccattc accacttgct cctaattgag 2520

ttgaaatctt tttacaacat agaaaactag aagatcgccc tttcttgctt catatatata 2580

gattttgtat catcgcaatt tcacatcaca cacacaagta aagcattagc aaccatagcc 2640

**CelA1 Promoter in C21 (GUS) and C30 (GFP) transgenic lines**

ID CesA1_Promoter;DNA; PstI-NcoI fragment; 2898 bp.

SQ Sequence 2899 BP; 971 A; 431 C; 440 G; 1057 t;

gacggcgcgc ctgaatcgga caacctcgtt gtctgtgata caactaaact tgtgaaggtc 60

tacactgttt cctaaaccaa agctgcggaa tcatcacgtg aggtttgtca tttattactg 120

tttctggagt atgttgttgc caaagattga catggcatcg ggagcactag gaaattatgt 180

aagcttgaaa tagttggtta tggtgagtat tatcgttgtt gaagaaaata caaaggaaat 240

aaatataatc ccatagctgt taccgtgttg atgtaaagtt gatactgcaa attttggagt 300

agttcattcc ttttaccttt catcccttgt gaacaagtaa gctgactcct ttcacttcat 360

tatacttagc attggactgc aaagcaaaaa taagtattgt agcatgataa acagaggtta 420

tgtaacccac acaaatataa tcatttattt gttttgctta aagggtcttt ttatttattt 480

aatattattt gaacatgatt ctcacgagaa aaataggtta aactctgctc tttgtgtaag 540

ttgtggattt aatttttata ctctaatttg gttagtttta gtccccatgc attttgaatt 600

aggcaatttt actcttatac ttttcaaaat ttgaaatttg gtttctctag ttgtgaattt 660

ggtttttatt ctccaatttg atcattatta gttatgcttt tcgaattttc agtgttaaat 720

tagatgataa cagttaaact tttaattaaa atagcgtggt ttttctatga atattatatg 780

aaaataataa tctcacataa catatgtttg cagtataaga ttttgaaaat ggctagaggt 840

gtagctagaa ggttgacggg gtcttgcccc caattgccaa gccaagaaat ttagagctca 900

aagtgtaatt ttactattat attaatttgt gatttcttaa aaattacagg gactgcataa 960

agaatttttc atttttggga caagtcacta cctgatccct ctccctcccc ttatttaaat 1020

ggaataataa caattttagt ccctctcaat tacaattaaa aatagtttaa ttcaactctt 1080

tttaaaacaa aatttttagc ttcactttcc caaactttta taattttatt ttgaccacca 1140

atactaaatt cttggcttca ctctttaaaa taatagaatt taacttaata aatttaaaat 1200

tattatttaa ttaagattga aattttaaag tttaaaaaag tataaataat aaaaatgacc 1260

aaattaagat ataaaaatta ggcatagatc aaaaattaag attgggattt gattaaaaaa 1320

tataagtctt caataaggct gaatagaata tgcatttttg ggtagcaaaa tcaatgtcag 1380

aaaacagagg tccatgacaa cagctacgtg gtagggacaa tctggtctgg ccaaattttg 1440

gtacatgttt ggtttggtgt cactcctctt ccattcctga ctcattatct taacctttct 1500

ttttcatttt ttaaatcata ataatcttaa tttgttgatt ataaacccag ttgaataatg 1560

tttatttgct cctttttcca atttatgatc tttgcttttc aacttactag atatgatatt 1620

tttcttctac tttttgaaat ctccaaatgt atgagacagt aaattaatgc gaggcctatt 1680

atttatggtt caacagtgac atacatttag acaagggtga aattatttat tagttactat 1740

actatgtgta aattacagat ttagtctata gacattaatt tgatattttt aatgtattta 1800

tttttcaaaa tttgaaattt cggtcttgac aagatagtga cggttaaatt tgttaagtta 1860

tgttattttc aagattttat gcgacatata tattatcaca tgtgttataa atacataggg 1920

atggatcttg acattgtgtt ttcttttgag gggtgattga ctaaaatttt taaaaatttt 1980

gaaggtttta atgagaattt ttaaacaatt ttgtatgtta aactaaaact ttcaaaaaaa 2040

attttgaaag gtttaatgag aattttaaaa attttgagcg ggctaattaa aatttttaaa 2100

aatgtataat aaaaaattca aaactctttg aggccataaa ggtcatcggg cccttaaata 2160

catcagcttg ttgtttcctc atattactca tgttatttca gttaacagat ataatggcta 2220

tcatttgatt taggagtgaa atctaaaaat tcgaaaagta taaaaactaa aaaggattaa 2280

attgaagaac attaattaaa tcaacaattt actattccaa taacagaatt ttgagttaac 2340

aaatttaact gctacaattt ggttcgagac caaaattaca aaacccgaaa agtattggga 2400

ctaaaattga tcaaattaga gtacatgggt taaattcaca acttacttat ggtacaagga 2460

ttaatagcat aatttctcct taggcaaatg ccagttagct aaagatgtta ccttgcccaa 2520

ccgaaagctt ccttaaactt cccggcaatt ttttaaattt ctttttccct tagaaaaaag 2580

accaaaatgt aagctttgct tgtcagagat ttctctgcaa atacattgac accaacaacc 2640

taccctccat tacactacca accggccttc cccttcaacc tttcttcacc attacaacat 2700

gcctatctcc acccttagcc caacatgcac ttatatcttg tgtttggttg tttttctttt 2760

tcatataaaa acacacacca agacacaaag gtattgagag gtaagtagag ggaaagaccc 2820

tttggttagc atattgtttg tagcattggg ttttttctca aggaagaaga aggagaaaga 2880

taagtacttt ttttgaga 2898

**Expansin promoter in C22 transgenic lines**

ID Expansin promoter; Not1-Nco1 fragment; DNA; 2192 bp.

SQ Sequence 2192 BP; 800 A; 335 C; 297 G; 760 t;

ggccgccagt gtgatggatg ggctggtatc catattccat ttttatatta tttattttta 60

atataattat tattttttta attattttaa aaattttaaa tttccattaa cctaattttt 120

ttctaaataa attattcata taaataaaga aattaaaaat actaacattt taaaaatctc 180

aaaaattcgt tattcgaaaa ttattgtttt ctgaaatata tatatgaaaa atactttaaa 240

attttatcaa ataatattta aaatatataa aataaagttc attttgtcaa cataaattta 300

gaaataaaaa atctaaaaat taatatgaaa aaaattgata accaaactaa actaaattat 360

aattaaaagt tcaaaaaatt aaaaaaatcc cgattgaacc agtatcaccc ctaaattaga 420

tgaggccata tttaacatat tagaaaatga aactctagaa aaatatataa aagtaaattt 480

attggcgaga gattagacaa agtcaatgca cccctcaatg aatagatatt attcccaatg 540

aaagtttcgt tttcaactct accaaaaact caaaagtctc aagagacgcg gcctgaatcg 600

tgactgggta gcgggtaaca atactacaac ccataaatgc tttatagcgc atagatcatg 660

ggtttagctt tggatcccat aaagtacaaa tactgaggtt tctttagtga atgatcatgc 720

atggacacat gatgtctctt tttaggcatt tgacaaactc gtcatttttt catacaattt 780

ctttggtcac ttaaattctc aataaaatta gaaaacagcc ctcaaaaata atattgatac 840

gttgaaagat ttatcaactt tcgaccatcg acactttaaa aaaattagaa agttataatt 900

tttttttcaa aaaactataa tacctctcta gttttagcta attaaattat tattatttta 960

ttattttatt gttattaaaa gtgtaacttg cactcaacta ttagtaagtt tacgttttga 1020

tcacttaatt tcagaaagtt aaaaaatggt ctttgaacta ttcgaaaatt ttcatttaag 1080

ttactggaat atttaaaagt ttttatttaa gtcaccgggc tattaagttt ttttttaaaa 1140

attcgattag caagttccaa gctacgattc gataagtgat acaatggatt tatacttatt 1200

gacaaataga atatacatta ggtccaagtt gatattacgg tcagtgttga aaatcgaaaa 1260

aaaatatttg gattttgatt cataaattta tgacttcaaa gctggttcat gaaaaagaac 1320

taaagtgtag gagggaagga aaaaaatatc ttttgattgg cacaaacagt gcgaacaaag 1380

aagaccacac aataacaatt ttaacaatat actaatttaa atgaaaaatt ttcaataatt 1440

taataagtta accgaggaaa acttactaag agttagttac ccctgttaaa ataactttca 1500

tgaagtaata gaaactttta gtacgtatca tcttatatag aacaatttct attttcagaa 1560

agtcaagaaa attgtattct agaaaatggc gacttcttca ccttcagtcc ttccctgatc 1620

ggcgcttgtg aaaaacgaaa aacctgagtc tgattggctg actgaaaatg aacctactca 1680

tcaccattca ctattaccaa cttcaaatga taggggaatt aactggtaaa gtgtaactcc 1740

accgatggtt gaggtggttg gctggagtta aatgagattt ttttagtttt gtttcaagtg 1800

gcttcaattg caagcaatta ggagactgcg ctggaataac ccctcgctca accttccgcc 1860

attgttatgg tttaattaaa cattatgttt ccatccatct atatttatat ccattaaaac 1920

aagtcgttga gcaaataatg gatactggat accatcatat ctatgattaa aattttgcat 1980

gtgccctttt aatgtatagc ttaagcctta attatcctcc aaatttgtac tctttcacca 2040

cttaattagc tacgtacggt acttagcgtt gcttgtcatc ttctgtacta caaactcttt 2100

ctcattttgt ataaatagct atacactttt tctctcctca aatcaataag gttaggtcag 2160

ccaattgttt gagctagctc ttactcccat gg 2192

**LTP promoter in C25 (GUS) and C31 (GFP) transgenic lines**

ID CFSP-LTP_Promoter; DNA; Pst1-Nco1 Fragment; 2983 bp.

SQ Sequence 2983 BP; 953 A; 493 C; 519 G; 1018 t;

ggaccgcgcc tcactataaa tttagcattt gatgtttata tattttatat aattgtcgat 60

aaaatgtcta aatgttatag actaaattta ttattatatg aataaaaata tgaacaattg 120

attagggtaa aagtattgtg gaagtcccat gtactaggag ttagattttg ccatttttac 180

taaaaaaatg ggcaaattaa tccttgtaca ttagattcaa aaagcaaatc ggtgcttttg 240

ttaaaaatcc catccaattc taccattaaa atcagtccat gtacgtcaaa atgaggtaca 300

catggcatat aatgtgtaac tgcctgatta ttctattcgc catgctggtt tttaacgata 360

gaaatagacg aaatttttaa caggaaatac caaattgctt ttttatctaa catacaggga 420

ctaatttgtc catttcttag taaaaaaggc aaaatgtaat ttgactcctg gtacacaagc 480

ttccagctcc atgcatggta attttatcga ttaattgtac tttcacttgg taatagctga 540

cttgtctata gatgttatat ttcacttaaa agcttgtagt gagtttcttg tacggggcga 600

agtcagaaaa tttgtttaga gtgtcgaaat taaattaaaa tttttaatag attatatctt 660

taaatttttt aaaggattaa atcgaacttt tattattttt aggaggatta aagtgtaatt 720

ttacttttac taattttaat ttttaaaatt tctaaaggac ctaaatgtat aattttccat 780

tttaggggtg ttgtttttaa ttttacattt ttcatgtcct ttgtgatagg tgaatattaa 840

ttaggaatca atcttattga aatcaggttt agatggaatt tcgattttgt gtgtgctttc 900

tttcaattga ataataatgc tcccccccac tgatatatat atatatataa atagttaacc 960

taaaaatttg cgtagtaagt aaacactata atttcttact tataaaaaga aaacttttct 1020

ttcatattaa tcatattacc tataactgca aagcattcat catcaatacc cataaatgct 1080

tacttataat aagaaaatat tttctttcat acaccaaatg ttagattgtt catccaccag 1140

taggaaacgt gagttggggt tagaccgtcg tgagacagat tagttttacc atattgatgg 1200

ttgtgttgca gtagtaattc aacatcctag tatgagagga accattgatt cgcacaattt 1260

ggtcatcacg cttgattgca aagccagtgg cttaaagcta ccgtgcactg gattatgact 1320

gaacgcctct aagtcagaat ttggattaga aacgatacac gcgtccgtcg cccgattgcc 1380

gaccctaatg atacatgtcg taggtttagc catctttatg aaagaaaagg ttttcttttt 1440

ataagtaagc aattataagg tttacttgca gatgcaaaat tttaggttaa ctattttagg 1500

agggggcatt atgattcaat tgaaagaaag ctggcacacg caaaataagg tttttatggc 1560

ccgtttagaa aatggatcag acctccagta aggatttttt ggtctgagcc caactcgaat 1620

ttacaaaaaa aaaattgttg ttgctgtttt tctactgttt tattgttcat ttcactataa 1680

tattgctatt tttttgttgt tattgtcaca cggtcgtgtg tcacacatgg gcatgtgccc 1740

taagtgtgtt gaaaaatagt ataggtgtag tttccataca ggccaacaca tggtcgtgtg 1800

tctcaagtca atgagttaca caaatagaga cacgggcagg gatacagtcg tgtgtcccaa 1860

cttcgaaagt cacacggtct ggggcattcc acacgaccat gtgtccccta tttccaggca 1920

ttttgagact tcaccctaaa cttctagagt tgtttcaaat tagcccctat ttgttcttaa 1980

atcattttag gatcttgtaa actcgtattt aggactaaat gtgtaatttt tactttaatt 2040

atgattgatt aattgattga tttgatagta atgcccgtga ccctaatccg ttagcgaaga 2100

ggggttaggg gttaggggtt ttattattat tttttagata ttgtataact cttgttttat 2160

ttttaatttt gttactactt caaaggcatt tgtttgtagt gttatttcga gtaggtttta 2220

tgggtgaaca acccttgacc gccaaatcaa tcacaagagt tcaacatttt atttattttg 2280

aaatgtatta aaaatcgtta atctatatat tcgccccatt attgggatta aatattcaca 2340

agggtttaga ccgtcatgag acagattagt tttatcttac tgatggtcac atcacaatag 2400

taattcaact taatacgaga ggaaccattg attcacgcaa ttggtcatcg cacttagttg 2460

aaaagctagg ggtgcgaagc taccgtacgc tggattatga ttgaacacct ctaagtcaga 2520

atccgaatta gaaacaatgc acgtgtccgt tgcctgattg ccaaccccaa taacacgtgt 2580

tgtaggttta accatgttta tgaaagataa ggtttttttt tttataagca agcaactata 2640

ggggtttact tccgtgcgca aatttttagg ttacctattt tgggaggggg gattatgatt 2700

caagtgaaag aaagttggca cacacacaat cagtacatct gttttgacag agacacagcc 2760

taaaaacagc agcaaacaag cctaaaggaa tcacccaaaa acaacaacca aaagtacaga 2820

ggaaaacaaa agaatccctg ttaccaccaa gctgaaaaaa agaaaataaa actcaacttt 2880

tggcaataaa aaccctccta ccctcaaccc ctaaccacgc aacaatcagc aatactccaa 2940

gcaaccattt tccttacaag tttgtttttc ttgtgattaa tcc 2983

**Rac13 promoter in C27 transgenic lines**

ID Rac13 promoter; DNA; Pst1-NcoI fragment; 4013 BP.

SQ Sequence 4013 BP; 1438 A; 704 C; 428 G; 1443 t;

ggacctagct actgacttaa gcccacggct aaatgacacg cctgtgtgtc aaagccgtgt 60

aatatttaga aggttactga gtttcttaca cggccataag gcacgccctt gtcccctaac 120

tgtgtgacac catgtaggct tgatttaagc caaattgcca ccccttttag aggtaatttt 180

cacaagccat attatataac atttatacct aaaaattttc aaccaaacaa ttatttcaaa 240

ccaaaacaag tatatttcat tttccaatca taaaacatca cattcaacat agtttgcata 300

cttaattatt catataatta cattactaaa tcaaatccta tacgtgccat gtaaaccaaa 360

aagttattta acaaaatcta ccggagtaaa tctggatagc gtgacccttg atgtagatcc 420

gatcctccgt atgtatataa gtcaatctac aaaacaaatc acacacacaa gtaagcttat 480

agaagcttag taagctcata ggcataataa cacatatctt acagaacact gtacacaatt 540

atatatgtat tagttaaact atttcatcct gcaaatcaca acttcattaa taaactcatt 600

tgaatgattt tcatgatgca attcacgaac ttaattcttt tgggcccatt tcttatttat 660

ttccattgtc aaattaggga acaataatgg agttgagtgc ttcattatca cattgccata 720

gtaaactatg gactttctca ttatcataca tcacacacca aagccatagc cctgccatag 780

tcttatacgg atcacatatc ataccgatgc catatcccag atatggtctt atacggaatc 840

acataatcac attataccga tgccatagcc cggttatggt cttatacgaa agcatatatc 900

atatcttttc cgtcaattca ttatggtcat aaaatgaaag cactcaaacc attgtttcaa 960

ctaatttgtt cttttagtta cacattgttc ttaagttaat acaatttgat aatattcatc 1020

tacaataaaa tacttacaat cataagattt tcatatcaaa cattgaactt ttccatatga 1080

acttacctgg gctaatttgc aaaagtcgta gaaattcagg aactattctt gaattttttc 1140

ttttccacga ttcatttcgt tttcttaatc tataattata aaatcattcc ttcattagca 1200

tctatttcaa ttccattcta tttcacaatt tatgccctta aattttcaaa attacacaat 1260

taccccaaac ttttcaattt ttacaattta gtccctaatt caatttattc atcaatcaaa 1320

ctaattcatt tcaaatatca ttttatctaa acactacaaa ctacttcaca gcctttgaca 1380

ttccaagctt caacacaaaa ccctaattcc aacaactttc acaattaggt cctaaaataa 1440

atttctatgc aaatctcttc ataaaatcat catataataa aattaaagcc ttaattccat 1500

gataaataat cataaaattt cagcacttac tcatggtaac tttctaaaat attcataaaa 1560

gcaaaaacta ttgaattaga tattaggacc taattgcaaa agtctcaaaa tcacaaaaat 1620

cacaataaat aatcaagaat tgagtttaca tgtaataaaa atatgagaga gcagcttaaa 1680

agaacccttc aatggtgttt tttctggaga agatgaagaa aattgaagaa aactagattt 1740

ttacctaata tatcatattt tacaatttaa tatttaccca attccaattt tgccccttgt 1800

ttacatttga tttttatttt ccaacacaca cctgccgtcc agctcaataa taggtgttta 1860

attccctatt tagtcctcat ttaataatta ttagagctat ttaatcacat ttcacaattt 1920

tgcacttaat tcaatttagt tctttttatc caattcacta tcgaaacctt aaaatttctt 1980

aacaaaactt taatactaac ttactaacat tccataaata tttataaaaa tatttatagc 2040

tcattttatg aattcaaggt ctcaatacct cgtttttatc ttatttattt ctacaaattt 2100

cttttaattc ataatttcac taattcaaaa ttatttctaa aacaacactt aaattttact 2160

tactaatgtc taaactcaca tgtcggattt agtgatctca aatcactatt ccgaatcact 2220

aaaatttggg tcgttacaat gctggtgtat gagaaccaat ttttaatagt aaaactaacc 2280

aatttttaat aataaaactg actcctagta caagagcttt tattcattct tctattttgc 2340

tttcctctag gcttggcaat cgagaatttt cttgtgttac aatataataa atacatcgta 2400

gaaataaatt ttattcaaat tgaagtctta accatcttta atatttgtag atgtaattta 2460

aatgaaagat aaatacatat tcttggacat gtattttcat cttaatgttt gtggctttgg 2520

tgataggtgt attgatgtac gatgtctttt aaatcacata tcacattttg agtttgtatg 2580

atgataagtc gacataaacg aaatatggtg tgatcttcac ttttgaactt tgataagtca 2640

ccaaacttta acaaagtttg attgtgtaca tatatatata tatcttcaaa ttttataata 2700

aaaattgtgt ttaaataatt tacagttata ttattttttt atctctaatt ttatttgtca 2760

ccaaattttt agttgatatt ttaacataaa aaaaattgta cacatttaca agcccatata 2820

caaataatta tataaatatt cattaaaaaa tatatttaaa tataggatat aaatataact 2880

attttagaat tattctactt taagataaca taggttaaat gtataattaa taaggttagt 2940

ttattgtaaa gatgagtata tatgtcgtaa acataatcac taaccatttt tattaacttc 3000

ttggttttga agtttcaaaa agaaaatgga agggaaattt gagagtaagt tcatgtttat 3060

attatacata atgaagttga tgttttcttc tttttaatat ttttatacaa aatatttaaa 3120

taaaataatt aaggattgaa tgaaaaatat aatgaaagtc gttttactaa tagtcatatt 3180

gcattttgtc gcatctactt aaataataga taaattaatt atggtacatt agatcaaaga 3240

acaaactaga ttttgtccca ttctattgtt aaaaactggt ccgtttacat taaaataagg 3300

tacatgttac atgccacgta taactatctg gttattctat caatcacgct aatttttaac 3360

agtagaaatg aatgtaattt ttaaatagaa agggtcaaat tgttatttga tctaacacgt 3420

agggattaat ttacttattt tcctaaagaa ataagtaaaa tataatttga atcttaatac 3480

aaaaactttc atgatacttt tatcatattt tacttataat ttaatattgt gagagtaaca 3540

aattaaaaaa catagaaaca ccaaaagtta gttatggtgt gactcatata cacagttaaa 3600

atttgaataa atttttttct tcgtcattaa ttccatcatg ggtttttttt tttctagtta 3660

agccataatt atcaaaataa tcatcattaa tcctatcaat accccgccct gcctccctcc 3720

ctcaatactt aaacccaact aacacccagc accaaacgca ctttaatagc cacctatttc 3780

tagccatgtc cttgcactta aagaaaagta aagctaacct gcaatcattc catatcgagg 3840

cctcaacaga taaagttggt tgatgggttt gcaccaagtt gttaaaaccc ggccctcaac 3900

ttcccttttc ttttcatcct ccccactcca caccctccaa ttttcttcat atggttctat 3960

tataagttct ttataatcac agaatcaaga taagtcctca gcaaacaaaa aac 4013

**Fb Late promoter (BamH1-NcoI, 4124 bp) in C33 transgenic lines**

**Fb Late promoter (Pst1-NcoI 2422 bp) in C34 and C32 transgenic lines**

ID Fb late(4-4)Promoter; DNA; BamH1-NcoI fragment; 4124 BP.

SQ Sequence 4124 BP; 1393 A; 552 C; 592 G; 1558 t; 29 other;

gatcccccgt ggactaaaca aaacatggga agatttgctg taaaaaaata aaagaagctt 60

actcaataac actttgtgaa ttgtatacaa aagactcaat gaaaaacaat aactcaatac 120

actttttttc actgatttac atcctttata taggctgaaa ctacaacaac tttagctaaa 180

aaaataggat aacctaatag caaaatcaca atcagatatt aaaccatgat tttagctaac 240

catttaacaa ctttattgaa actaatttga atatttcatc tgctgatatg cccaagattt 300

taggccacta accgatttgg tggtgaactt taacatgtca tgcatttgta actgtttgaa 360

acaagttttt tgcattattt tactatatga actgtttgat taggttgagt tacacactga 420

gcttgtaagc tcactcaaat ttttctaatt tctaaggtga tcagcaaact taggaccggg 480

cggcgtacga gagctcggat tgattttcta gttaataaat aagacgattt atgtttttaa 540

actattatgg actttttgga ctatgtaact gtttgggact ttatttttgt tttttatttg 600

ctttttttgg atttagtaat tattattttt aaactgcaaa attatatgtt tttacaaact 660

aagtcacagt tttcaaaatt ccataactta gaatttttcg ctgcaaaata aagtaatcat 720

ttaagtgttt tttctgtaat aaaataaata aataatttta acgagtattt tcctaaaaat 780

tggaaattga tttaccaaaa ttagtatgtc aaaacacatg tttatatgtt acaggggcga 840

tatcgtctag gcaaataaca tctaggcggg gtttggagtg ttacagggcg agtgggctca 900

ttttgagtaa gtatagttag ggccgagttt tagattgcat attcaaggtc aaagattttg 960

taaacttcga tgaatgatat gtatgattgt ccgattaacg aaatatgttt ttttcttttg 1020

tgtgtgtttt atctcgtgtg ataagtatat agtatgtttt attccaattc ttatggcatg 1080

tgacattgtg gctattctaa ttaaattgat ttgttattat tgaaatctga tgcatctgtt 1140

ctacaaagca tggaatctca tgcctactgc tttctgttaa agatacgatt gcaagtttaa 1200

catgcttact atttgatttt gtccttgcat gctatgtcac attacatggg gttgggatga 1260

tatggtaagg aggaagtttt gacagtttaa tgatttgcac tatctggtgg tttaaccaca 1320

tatttcttat ggcatcttga ctgcggttat ggtggctcga ccgcccatat ctgttctgga 1380

aatttatctg tgactctggt ggcattgtct acattatttg ttggtgtgtt ttggatggac 1440

gagtcgtggg gaactctatt tggtgtgttg cggagttggg taggaaattt tcgaaaaaaa 1500

tttgcattgt gtttttctga aaaatattgc attaacataa tcatgcattc tcaattttgg 1560

tcaattgaac gttataaaat tctctatgat atcctgatct gtttattaca ttatatgtgt 1620

ttatatgtgt ttatgcttga gttaagtcaa acattgagat tcatagctca cccaattatt 1680

taatcatttc aggcaatctg cagacttagg attggatggc gttcaggagc ttggattggt 1740

tttctcacat catattttat taaataatta ttaattaaaa tttatggact tttggactgt 1800

ctgactaatt ttcagaattt tattttggtt ttgggttttg ttgarttttt tagataatta 1860

ttttaaatat tctgcataat ttttctgtta tttgaaaagg atgttcgaat tttttttcaa 1920

aattgaaacg tttaagaatt tttactactg caaattcaga ataagtgaat ttgtttttta 1980

gaaagattaa ataagttagt attacgattt ttagtttgat ttggtggaaa gtaatgtatg 2040

tttttgaaca taattatttg acaataatta agttttctag graataaacg gaaatatctt 2100

ctttyttttt tgtaaaatta ctaatgcaag aacaaacaac gttttgggra gcaaataatc 2160

tagctttaag tagtcagtgt aactctcaaa atctggtcat aacttctagg ctgagtttgc 2220

tgtgctacag tagtaagtct atagaaactt acctgacaaa acgacatgac gtcagggtcg 2280

aatctacaac ttttcctttt tcttcaatta acatatggtt gattcaagtt ccgatctata 2340

ataatttatt acgatttatc aatttcaatt accttatatc atcctattat aaatataagt 2400

cagttcaatt cagttttcga aagttcccwa aaattttsaa ttttattaaa tttattccct 2460

aaaaccgaaa tagtkatatc tttcaaattt aagtttcatt tttcaatccg atttcaattt 2520

catcctttta taactctcta tkatctataa ttacataaat ttcaaaytaa ttttgaaata 2580

twtacacttt agtccctaag ttcaaaacta taaattttca ctttagaaat taatcatttt 2640

tcacatctaa gcatcaaatt taaccaaatg acacaaattt catgattagt tagatcaagc 2700

ttttgagtct tcaaaaacat aaaaattaca aaaaaaaaaa aacaaactta aaatcattta 2760

tcaatttgaa caacaaagct tggccgaatg ctaagagctt aaaaatggct tcttttgttt 2820

ctttttgttg caaacggtgg agagaagagg gaaatgaaga ttgaccatat ttttttatta 2880

tgttttaaca tataatatta ataatttaat cataattata ctttggtgaa tgtgacagtg 2940

gggagatacg taaagtattw taacattata ctttttgcaa gcagttggct ggtctaycca 3000

agagtgatca aagtttgagc tgccttcaat gagccaattt ttgcccataa tggataaagg 3060

caatttgttt agttcaactg ctcacagaat aatgttaaaa tgaaattaaa ataaggtggc 3120

ctggtcacac acacacaaaa aaaaaactaa tgttggttgg ttgaatttta tattacggaa 3180

tgtaatrtta tattttaaaa taaaattatg ttatttakat tcttaatatt ttggagcatt 3240

ccatactata atytcgtata cataatatta aaatatagta atataaagtg taattaactt 3300

taaattacaa gcataatatt aaattttgaa tcaattaatt tttatttcta ttattttaat 3360

taatttagtc tattttttca aaataaaatt taaatctaaa taaaaataat ttttccttaa 3420

tgttgaaaca actcatgtta tacttcaaaa ttataagtat tatatttacc ttgatgattt 3480

atttattagt atattaattc tgattataat tatggtggga tacaatcgct ttccactawa 3540

tattttaact atgatttatw aatttatttc aacatcgtaw atttacttat taatacataa 3600

tttatcataa ttttatggaa attgagacca agaaacatta agagaacaaa ttctataaca 3660

aagacaattt agawaaaaat gtacttttag gtaattttaa gtactcttaa ccaaacacaa 3720

aaattcaaat caaatgaacy aaataagata atataacata crgaayatcy tacttgtawt 3780

cttacattcc crtaatywta ttatgaaaar taatmttata ttactcgaac taaatgttgt 3840

cacaaattat tatctaaata aagaaaaaca cttaattttt ataacatttt ttcatatatt 3900

tgaaagatta tattttgtat atttacgtaa aaatatttga catagattga gcaccttctt 3960

aacataatcc caccataagt caagtatgta gatgagaaat tggtacaaac aacgtggggc 4020

caaatcccac caaaccatct ctcattctct cctataaaag gcttgctaca catagacaac 4080

aatccacaca caaatacacg ttcttttctt tctatttgat taac 4124

//
